# Metabolomics based on GC-MS revealed hub metabolites of pecan seeds germinating at different temperatures

**DOI:** 10.1186/s12870-023-04209-8

**Published:** 2023-04-10

**Authors:** Tingting Xue, Sian Liu, Jia Liu, Yingdan Yuan

**Affiliations:** 1 Department of Civil and Architecture and Engineering, Chuzhou University, Anhui, 239000 China; 2 College of Horticulture and Landscape Architecture, Yangzhou University, Yangzhou, 225009 China

**Keywords:** Pecan seeds, Metabolome, WGCNA, Amino acids

## Abstract

**Background:**

As an important plant source of food and edible oils, pecans are rich in metabolites. Few studies have focused on metabolites involved in pecan seed germination at different temperatures.

**Results:**

In our study, we germinated pecan seeds at different temperatures and found that, the germination rate and water content were highest at 30°C. It was found that the radicle of pecan seeds could sense seed coat cracking by observing the microstructure and cell ultra-structure of the seeds at the early stage of germination. We compared the metabolomes of seeds at different temperatures with different germination processes. A total of 349 metabolites were identified, including 138 primary metabolites and 211 secondary metabolites. KEGG enrichment analysis indicated that the differential metabolites were mainly enriched in the metabolic pathways, amino acid synthesis pathways and ABC transporters. Using weighted gene co-expression network analysis (WGCNA), three modules of closely related metabolites were identified. In the brown module, most of hub metabolites were amino substances, whereas in the blue module, many hub metabolites were sugars.

**Conclusions:**

Amino acids and carbohydrates play an important role in pecan seed germination. Differential metaboliteanalysis showed that 30°C was the temperature at which metabolites differed most significantly. This study provides useful information for further research on the seedling establishment of pecan seeds.

**Supplementary Information:**

The online version contains supplementary material available at 10.1186/s12870-023-04209-8.

## Introduction

*Carya illinoensis* Koch is native to northern United States and Mexico. It is a tall Hickory tree belonging to the genus Carya in the family Juglandaceae [1]. The seeds of pecan have high-temperature germination characteristics, which are relatively rare and have been considered to be the main reason for the low germination rate of this plant [2]. High temperatures increase amylase activity in pecan seeds and promoted seed germination. The metabolism of sugars and proteins plays an important role in kernel response to temperature changes [3].

Temperature is recognized as one of the most important environmental factors that affectseed germination [4]. First, environmental temperature can directly or indirectly affect the germination of plant seeds, and suitable temperature conditions can break the dormancy of dormant seeds thereby, indirectly affecting seed germination time. For some non-dormant or broken dormant plant seeds, a suitable temperature can directly induce germination [5]. Second, temperature fluctuations are of great significance to plant seed germination, and plant seeds with different germination strategies have different sensitivities to temperature fluctuations, resulting in differences in germination behavior [6]. At present, there are many studies on the effects of temperature on seed germination, and the results are even more varied. Yang et al. [7] found that the germination rate of *Primula beesiana* seeds without other treatments was significantly higher at 25/15°C than at 15/10°C, indicating that higher temperatures increased the germination rate of *P. beesiana* seeds. *Polygonum persicaria* seeds were buried in soil and dug monthly for germination experiments over 3 years by Bouwmeester et al. [8]. The results of their study showed that soil temperature has a certain correlation with the annual cycle of seed dormancy and that the germination rate at 30°C was higher than that at 10 and 20°C. Thompson studied the responses of 112 weed species in Sheffield to temperature fluctuations, and the results showed that most plant seeds required large diurnal temperature fluctuations (> 5°C). In contrast, some plant seeds can germinate at a constant temperature, and some plants still require less diurnal temperature fluctuations for seed germination. It can be seen that temperature has a great influence on seed germination, and the germination temperature is of species specific.

During plant growth and development, the temperature directly affects the vitality of seeds, and the appropriate temperature range is conducive to seed storage andsubsequent growth and development [9, 10]. Metabolomic studies have examined changes in the types and quantities of metabolites in organisms, as well as their metabolic pathways and metabolic networks [9]. Metabolomics can be used to observe changes in plant metabolites of plants after they are subjected to various internal and external environmental stresses (gene changes or environmental changes) and to judge how to improve tolerance to adversity or adaptability [11]. The normal germination and growth processed of seeds involve various metabolic changes, such as changes in the nutrients stored inside the seeds, proteins and carbohydrates, which provide the seeds with the energy required for germination and growth [12]. During the germination process, the pentose phosphate pathway is converted from the tricarboxylic acid cycle pathway or the glycolysis pathway. The corresponding oxidation reaction occurs when the seed changes from a dormant state to a germination state, resulting in a large amount of metabolism and reactive oxygen species [13]. Gu et al. [14] used GC-MS and LC-MS techniques to study and analyze soybean metabolites at different germination times. A total of 58 metabolites were screened, including macromolecular derivatives, amino acids, inositol metabolites, phytosterols, antioxidants, isoflavones, and soy saponins. Metabolomics analysis can reveal the response pathways initiated by seeds stored at different temperatures and the expression of important related substances, laying the foundation for future variety breeding.

Seed germination is an important stage in the continuation and development of races, and is an important method of plant cultivation and production. Pecan seeds have a less common high-temperature germination trait while having a hard shell. In our previous study, we found that the inclusions of pecan seeds at different temperatures changed the early stage of germination, which provided a basis for further exploration of the high-temperature germination mechanism of pecan seeds [15]. Therefore, in this study, three different temperatures (15, 25, and 30 °C) were selected for germination experiments to study the effects of different temperatures on the germination of pecan seeds. In this study, the germination rate and water content of pecan seeds germinated at different temperatures were compared, and the microscopic structure and cell ultrastructure of the seeds at the early stage of germination were observed using paraffin section and staining. Metabolomics was used to analyze the differences in metabolites and enrichment pathways of differential metabolites in the seeds of pecans at different temperatures. Finally, weighted gene co-expression network analysis (WGCNA) was used to identify the characteristic metabolites of pecan seeds at different temperatures. This study is of great significance for understanding seed germination and the large-scale cultivation and seedling rearing in pecan.

## Materials and methods

### Plant materials and experimental design

Fresh pecan seeds, used in this experiment were provided by the Horticultural Institute of Jiangsu Academy of Agricultural Sciences, China in October 2016, and the cultivar is “Jinhua”. The seeds were stored at room temperature in the Southern Forestry Seed Inspection Center of the State Forestry and Grassland Administration (Nanjing Forestry University Campus). 3 × 50 seeds were sampled when pecan seeds were imbibed at room temperature for 0, 5, and 10 days. After imbibition for 10 days, the seeds were placed in a germination box covered with moist absorbent cotton and placed in a constant temperature full light incubator at 15, 25, and 30°C for germination. On the 2nd, 4th, 6th, and 12th days (days from the time of water absorption, sampling was recorded as 12, 14, 16 and 22 days). Seeds with unbroken shells were used as test samples and 3 × 50 seeds were sampled. When sampling all samples, the germination rate was counted, the seed husks were removed, the seed kernels were chopped and mixed evenly, and 3 × 5 g of seed kernels were collected using a constant temperature drying method (drying at 103°C for 24 h) to determine the water content of the samples. The remaining samples were stored in a -80 °C refrigerator for metabolomic analysis.

### Paraffin section and staining of seed in early germination

We soaked 100 experimental pecan seeds at room temperature (ranging from 15 to 25°C at room temperature) for 10 days by placing them on a cotton bed followed by culturing in a constant temperature incubator at 30°C. Dry seeds were removed and prepared for imbibition (1, 4, 8, and 15 days). Five seeds were selected when the seed shell cracked, and the radicle was exposed. The seed shell was peeled off and the seeds containing the radicle end were cut and prepared for sectioning. After separation from the remaining tissues (hypocotyl and cotyledons), paraffin sections were prepared and stained using the following method.

Tissue morphology and structure of the samples (hypocotyls and cotyledons) were examined using the paraffin section method. The collected samples were cut, fixed in fixative solution, decolorized with ethanol, and embedded in paraffin wax blocks. The paraffin blocks were sectioned, rehydrated using ethanol, and washed with water. Safranin O staining was performed by immersing the sections in Safranin O for 2 h. The sample was decolorized using gradient ethanol and fixed in plant solid green staining solution. The tissue sections were mounted with neutral balsam and observed under a microscope. Panoramic scanning was performed using a Pannoramic 250/MIDI scanner (3D HISTECH, Budapest, Hungary) after placing coverslips.

### Ultrastructure of seed kernel cells in early germination

We soaked 100 pecan seeds at room temperature (15–25°C) for 10 days by placing them on a cotton bed and culturing them at a constant temperature incubator at 30 °C. The dry seeds on the 5th, 10th, and 15th days after imbibition, as well as 5 seeds with cracked seed shells and exposed radicles, were collected. The seed coat was peeled off and the radicle (tissue in the middle) and cotyledons (tissue in the center of the right half of the midseed suture) were removed. Transmission electron microscopy (TEM; Hitachi H-600, Tokyo, Japan) was used to examine the morphologies of the prepared samples. The detailed process is the same as that described in a previous study [16].

### Sample extraction and GC-MS metabolome analysis

The sample 100 mg of sample was accurately weighed and transferred to a 1.5 mL Eppendorf tube. Two small steel balls were added to each tube. The internal standard was prepared by dissolving 360 µL of cold methanol and 40 µL of 2 chloro-l-phenylalanine (0.3 mg/mL) in methanol. This solution was added to each sample and the samples were incubator at 80°C for 2 min. The samples were then ground at 60 HZ for 2 min. The mixtures were ultrasonicated at ambient temperature for 30 min. Thereafter, 200 µL of chloroform was added to the samples and the mixtures were vortexed by adding 400 µL of water. The samples were vortexed, and ultrasonicated at ambient temperature for 30 min. The samples were centrifuged at 12,000 rpm at 4°C for 10 min. In a glass vial 500 µL of supernatant was collected and dried in a freeze- concentration centrifugal dryer. QC samples were prepared by mixing aliquots of all the samples to form a pooled sample. Each QC sample had the same volume as that of the other samples. Subsequently, 80 µL of 15 mg/mL methoxylamine hydrochloride in pyridine was added. The resulting mixture was vortexed vigorously for 2 min and then incubated at 37°C for 90 min. Then, 80 µL of BSTFA (with 1% TMCS) and 20 µL n-hexane were added to the mixture, which was vortexed vigorously for 2 min and derivatized at 70°C for 60 min. The samples were placed at ambient temperature for 30 min before GC-MS analysis.

The derivatized samples were analyzed using an Agilent 7890B gas chromatography system coupled with an Agilent 5977 A MSD system (Agilent, Santa Clara, CA, USA). A DB 5MS fused silica capillary column (30 m × 0.25 mm × 0.25 μm, Agilent J & W Scientific, Folsom, CA, USA) was used to separate the derivatives. Helium (> 99.999%) was used as the carrier gas at a constant flow rate of 1 mL/min through the column. The injector temperature was maintained at 260°C. The injection volume was 1 µL in the split mode (split ratio 2:1). The initial oven temperature was 60°C, ramped to 125°C at a rate of 8°C/min, to 210°C at a rate of 4°C/min, 270°C at a rate of 5°C/min, 305°C at a rate of 10°C/min, and finally held at 305°C for 3 min. The temperatures of the MS quadrupole and the ion source (electron impact) were set to 150 and 230°C, respectively. The collision energy was set to 70 eV. Mass data were acquired in full scan mode (m/z 50–450), and the solvent delay time was set to 5 min. The QC samples were injected at regular intervals (every 10 samples) throughout the analytical run to provide a set of data from which repeatability could be assessed [17].

### Data processing and statistical analysis

The acquired MS data from GC-MS were analyzed using the Chroma TOF software (v 4.34, LECO, St Joseph, MI). Metabolites were analyzed qualitatively using the NIST and Fiehn databases, which were linked to the Chroma TOF software. Briefly, after alignment with the Statistic Compare component, the CSV file was obtained with three-dimensional data sets including sample information, peak name, retention time m/z and peak intensities. The detection table peaks of the samples in GC-MS were 1336 in total, and the internal standard was used for data quality control (reproducibility). After internal standards and any known pseudo positive peaks, such as peaks caused by noise, column bleed and BSTFA derivatization procedure, were removed from the data set, and the peaks from the same metabolite were combined, the detectable metabolites of silkworm hemolymph samples in GC/MS were 361 in total. The resulting data were normalized to the total peak area of each sample, multiplied by 10,000.

The differences between the metabolites of the two treatments were maximized with Orthogonal projections to latent structures-Discriminant Analysis (OPLS-DA) to identify differential metabolites. Based on the OPLS-DA results, preliminary screening of differential metabolites was performed using the obtained Variable Importance in Projection (VIP) of the OPLS-DA model for multivariate analysis. In our study, we selected VIP ≥ 1, fold change ≥ 2, or fold change ≤ 0.5, as the differential metabolites for the next step of analysis [18]. Using the R program, we used principal component analysis (PCA) to visualize the buildup of pecan seeds metabolites at different temperatures and germination processes. Statistical analysis was performed using the Statistical Package for the Social Sciences software (SPSS) version 22.0 software (IBM SPSS Inc., Chicago, IL, USA).

### WGCNA analysis

The key modules and hub metabolites related to germination temperature and stage in pecan seeds were analyzed using WGCNA. The weighted gene co-expression network analysis (WGCNA) was performed using the WGCNA R package (v. 1.68) [19]. The correlation between the module and major metabolites content was calculated using the calculated values of the relationship between the module characteristic metabolites and Pearson’s correlation coefficient. The results of WGCNA were imported into the Cytoscape software (Version 3.9.1, Cytoscape Consortium, Bethesda, MA, USA) to obtain metabolite network diagrams for each module.

## Results and discussion

### Seed germination rate and water content

The water contents of pecan seeds soaked at room temperature for 0, 5, and 10 days were 4%, 23%, and 26%, respectively. Table S1 shows the changes in water content and germination rate during seed culture. The water content of the kernels of dried pecan seeds was only 4%. After absorbing water for 10 days, the water content of the seeds was stable at 25–26%. Until seed germination began, the water content increased further and gradually stabilized at approximately 38%. Within 30 days, pecan seeds did not germinate at 15℃, only 4% germinated at a later stage at 25℃. The seeds were cultured at 30°C and germinated after 14 days (i.e., placed in a 30°C incubator for 4 days), and the germination rate was 21%. By the 22nd day, the germination rate had reached 100% (Supplementary Table S1).

From our results, it can be seen that pecan seeds germinate best at 30°C, which is a typical high-temperature germination temperature. Temperature not only affects plant growth and flowering, but also plays an important role in seed dormancy and germination [20]. Temperature can induce or release seed dormancy, and seeds of some species require a specific temperature range for germination, which often enhances dormancy and may even induce dormancy in hydrated seeds. However, dormancy of relatively dry seeds gradually releases with temperature changes [21, 22]. High temperatures can promote the germination of some seeds and high-temperature stratification can promote the release of seed dormancy in hornbeam plants [23]. In some cases, low temperatures may also induce dormancy, and seed dormancy is greatly affected by temperature, especially in the range of 1–15°C [24].

### Microstructure and cell ultrastructure in the initial stage of seed germination

**Fig. 1:**
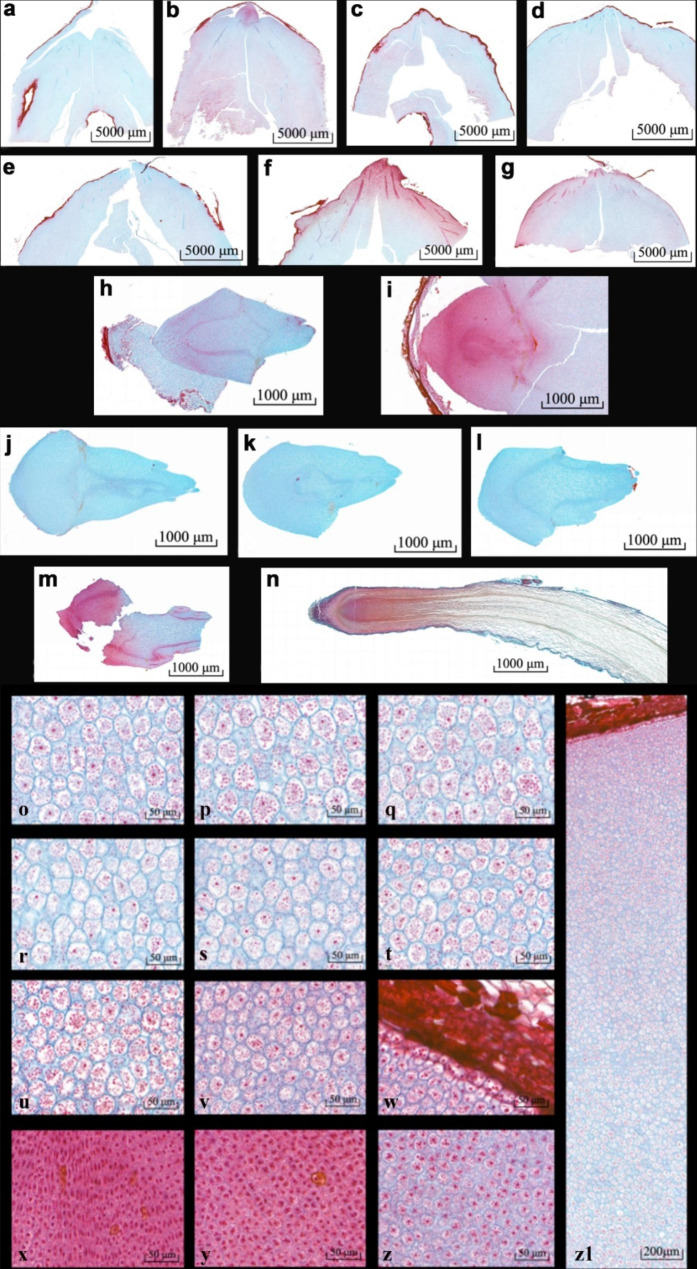
Micrograph of pecan seed section during germination stained with safranin and fast green. (**a-g**) Paraffin sections of hypocotyls and cotyledons. a: dry seeds; b: imbibition 1 day; c: imbibition 4 days; d: imbibition 8 days; e: imbibition 15d; f: seeds with cracked seed coat; g: seeds with radicle breaking through the seed coat. (**h-n**) Paraffin sections of radicles. h: dry seeds; i: imbibition 1 day; j: imbibition 4 days; k: imbibition 8 days; l: imbibition 15d; m: seeds with cracked seed coat; n: seeds with radicle breaking through the seed coat. (**o-z1**) Safranin-fast green counter-stained photomicrographs of paraffin sections of the radicle, hypocotyl, and cotyledons of pecan nuts seeds imbibed for 1 day. (**o-w**) continuous tissue from the cotyledon to the hypocotyl to the seed coat; (**x-z**) tissue from the center to the edge of the radicle; z1: the entire tissue from the cotyledon to the hypocotyl to the seed coat

In the microscopic section, the tissues stained by safranine are generally nuclei, chromosomes, proteins, vascular bundles, and plant lignified, corky, and keratinized tissues, whereas the tissues stained by fast green are mostly cellulose. As shown in the figure, the radicle and hypocotyl of dry seeds (Fig. 1a and h) and seeds that had been imbibed for 1 day (Fig. 1b and i) were still difficult to separate, and the radicle and hypocotyl could be completely separated after 4 days of seed imbibition. The hypocotyls of the pecan seeds were located on both sides of the radicle. The sections (Fig. 1a–g) showed that there was no clear boundary between the cotyledons and the hypocotyl. Hypocotyl elongation was observed when the radicle was elongated for 1–2 days. In addition, the radicles were stained with safranine-fast green, and the hypocotyls showed a large area of ​​red staining at the three time points of seed imbibition for 1 day, seed shell cracking, and radicles breaking through the seed coat (Fig. 1b, f, g and o–z1). However, after dehiscence of the seed shell (Fig. 1b, f and g), the hypocotyls and cotyledons showed different staining patterns, with the hypocotyl showing a large area of ​​red and green cotyledons. The hypocotyl cells near the seed coat were closely arranged, and the number of cells gradually increased and became increasingly loosely arranged from the hypocotyl to the cotyledon area (Fig. 1o–z1). Meanwhile, elongation of the seed radicle appeared sometime after dehiscence of the seed shell (Fig. 1j-n).

**Fig. 2:**
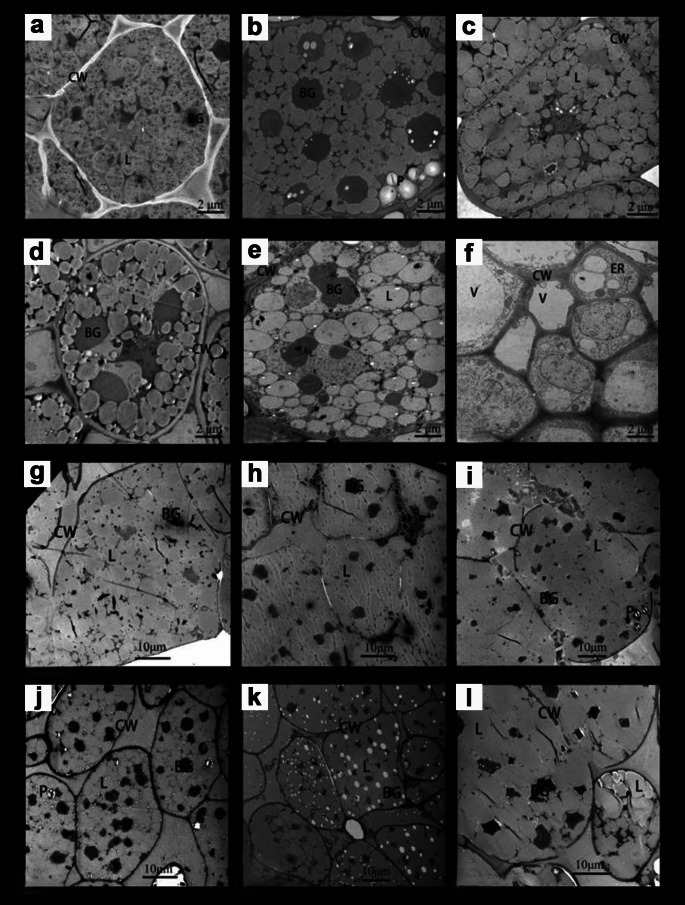
Cell structure of pecan seed during germination. (**a-f**) Radicle. a: dry seed; b: imbibition for 5 days; c: imbibition for 10 days; d: imbibition for 15 days; CW: cell wall; BG: highly electron-dense material; L: lipid droplet; V: vacuole; P: amyloid; ER: endoplasmic reticulum. (**g-l**) Cotyledons. g: dry seeds; h: imbibition for 5 days; i: imbibition for 10 days; j: imbibition for 15 days; k: seeds with cracked seed coat; l: seeds with radicle breaking through the seed coat; CW: cell wall; BG: highly electron-dense substance; L: lipid droplet; P: amyloid

The distribution of the ultrastructure of cells in the radicle and cotyledons during the initial stage of pecan seed germination is shown in Fig. 2. Before the radicle broke through the seed coat, only a large number of lipid droplets and highly electron-dense substances were observed in the radicle and cotyledon cells (Fig. 2a-e and g-k). The cells also contained a small number of amyloplasts, which were few in number and unevenly distributed (Fig. 2i and j). The radicle cells were 8–12 μm in size, while the cotyledon cells were 10–40 μm in size. When the radicle of the pecan seeds broke through the seed coat, the cells of the cotyledons did not change significantly (Fig. 2l), whereas the ultrastructure of the radicle cells changed significantly (Fig. 2f). At this time, various organelles, such as the endoplasmic reticulum appeared in the radicle cells, among which the central large vacuole began to appear in the cells (Fig. 2f). The size of the radicle cells was only 2–4 μm, indicating that the radicle was underwent cell division.

The radicle of the shelled pecan seed can sense capable of sensing the cracking of the seed coat. There is no chemical inhibition of seed germination by the seed coat, and the seed coat restricts the germination of pecan seeds because of its mechanical restraint of the seed coat. Through the study of paraffin section and transmission electron microscopy, it was found that elongation and division of radicle cells occurred after cracking of the seed coat. The radicle can elongate only when the seed coat is split; the pressure produced by the seed coat on both sides of the cotyledon disappears, and a small part of the seed coat covering the top of the radicle cannot directly hinder the elongation of the radicle.

### Metabolomic profiling and principal component analysis

To study the variations in metabolites during the germination of pecan seeds at different temperatures, we performed GC-MS metabolic profiling of different samples. According to the metabolomic results of pecan seeds germinated at different temperatures, 349 metabolites were successfully identified, including 138 primary metabolites and 211 secondary metabolites (Supplementary file Table S2–S4). The primary metabolites include: 20 amino acids and 14 nucleic acids, 16 fatty acids, 3 peptides, 17 esters, 57 carbohydrates and their derivatives, and 9 organic acids and 2 kinds of others; secondary metabolites include: 9 simple phenols, 8 steroids, 5 flavonoids, 1 quinone, 6 terpenoids, 44 non-protein amino acids, 1 cyanogenic glycoside, 27 alkaloids, 15 amines, 31 other nitrogen-containing compounds, 47 organic acids species, and 17 other secondary metabolites.

**Fig. 3:**
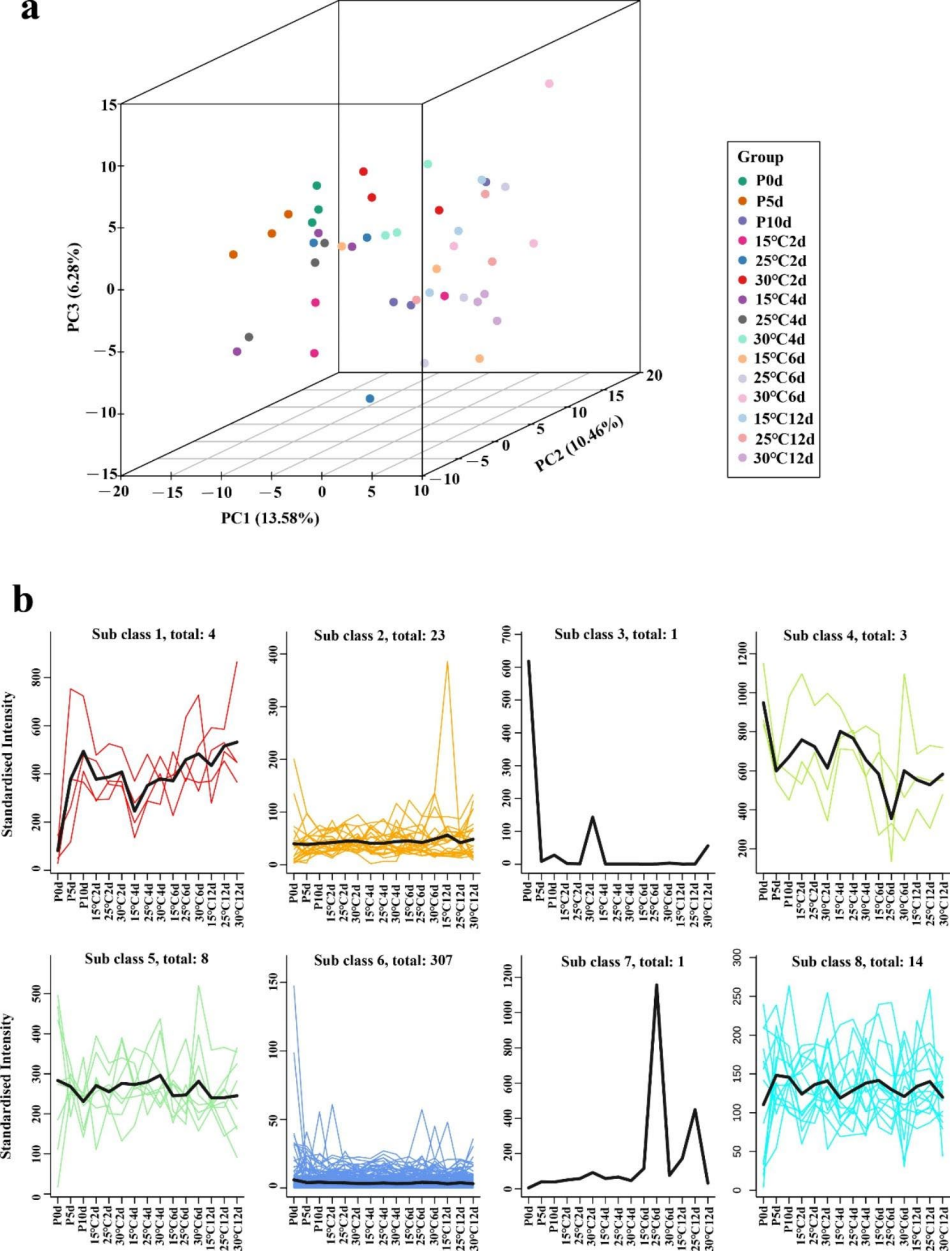
Metabolomic analysis of pecan seeds at different temperatures. (**a**) 3D-PCA score plots for all samples; (**b**) The K-means analysis of all metabolites

We performed a principal component analysis (PCA) of the data to obtain a prior understanding of the overall metabolome (Fig. 3a). PC1, PC2 and PC3 explained 13.58%, 10.46% and 6.28% of metabolite variation among all samples, respectively. Most of the samples could still be distinguished, but some samples could not be distinguished because the sampling time for seed germination was relatively frequent and the metabolites were relatively similar. The K-means of all metabolites was studied to investigate trends in the relative content of metabolites in different subgroups (Fig. 3b). The metabolites were classified into eight clusters, of which, subclass 6 contained the largest number of metabolites, including primary metabolites: amino acids and sugars. It can be seen from this that amino acids and sugars are the most important metabolites during the germination of pecan seeds.

### Differential accumulated metabolites analysis

**Fig. 4:**
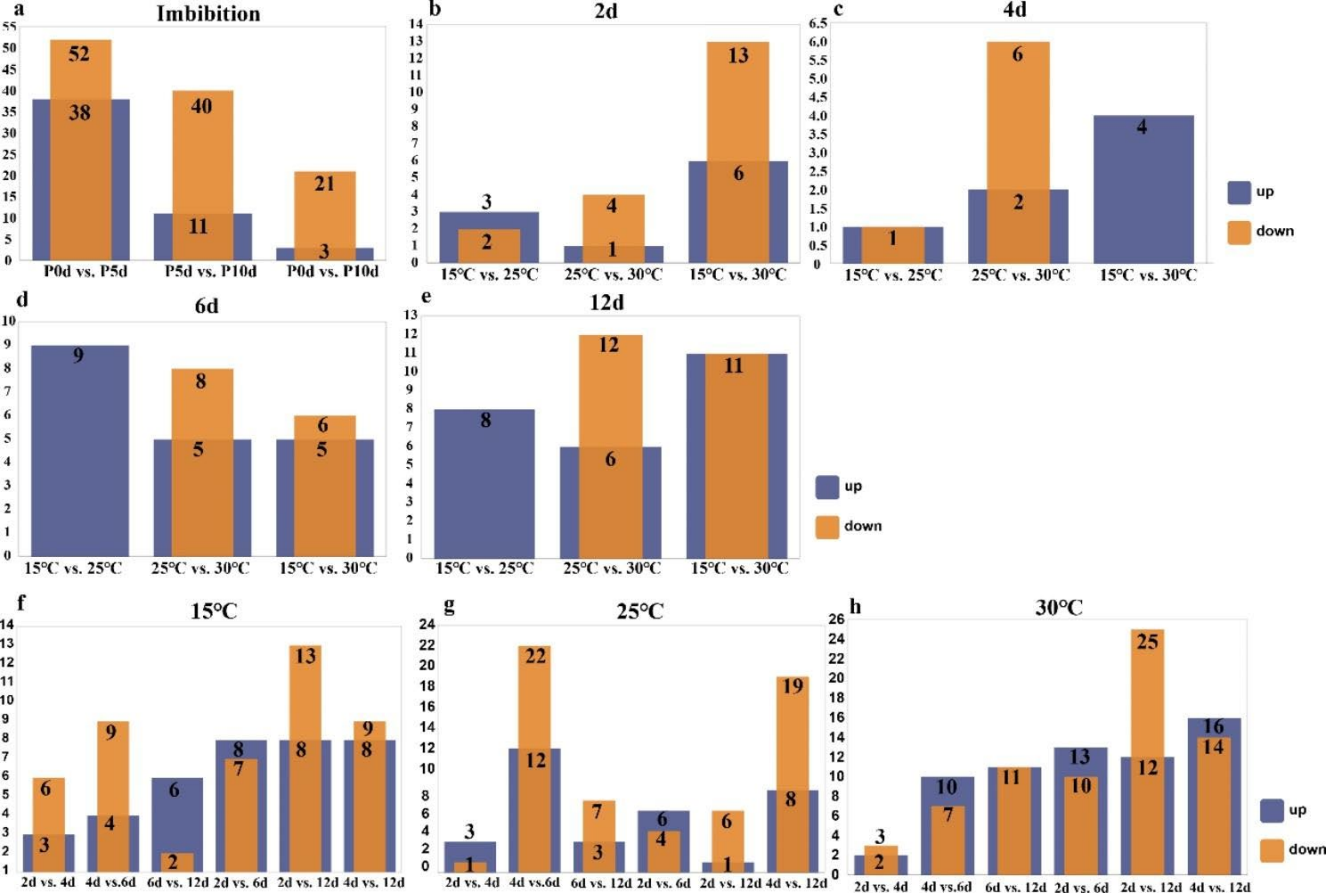
Number of DAMs. (**a**) Imbibition; (**b**) 2d; (**c**) 4d; (**d**) 6d; (**e**) 12d; (**f**) 15℃; (**g**) 25℃; (**h**) 30℃

To gain insight into the variance of metabolites among the different temperatures of pecan seeds, differential accumulated metabolites (DAMs) were identified using a fold-change ≥ 2 or ≤ 0.5 and a VIP ≥ 1 between pairwise comparisons (Fig. 4). There were 90, 51, and 24 DAMs detected in the imbibition group (P0d vs. P5d, P5d vs. P10d, and P0d vs. P10d, respectively) (Fig. 4a). The most up-regulated and down-regulated DAMs were identified in P0d vs. P5d, whereas the least up-regulated and down-regulated DAMs were found in P0d vs. P10d. It can be seen that from the beginning of imbibition, up to 5 days, is the stage with the most significant difference in metabolites. Additionally, we examined the variation in DAM in pecan seeds at different temperatures and during different germination processes (Fig. 4b-h). Among the different germination processes, the greatest number of DAMs (22) was detected in 15℃ vs. 30℃ of 12days, and the smallest number of DAMs (2) was detected in 15℃ vs. 25℃ of 4days (Fig. 4b-e). Among different temperatures, the greatest number of up-regulated DAMs (16) was detected in 4 days vs. 12 days of 30℃, and the greatest number of down-regulated DAMs (25) was detected in 2 days vs. 12 days of 30℃. Therefore, 30℃ is the temperature at which metabolites differ most significantly during the germination of pecan seeds.

### KEGG enrichment

**Fig. 5:**
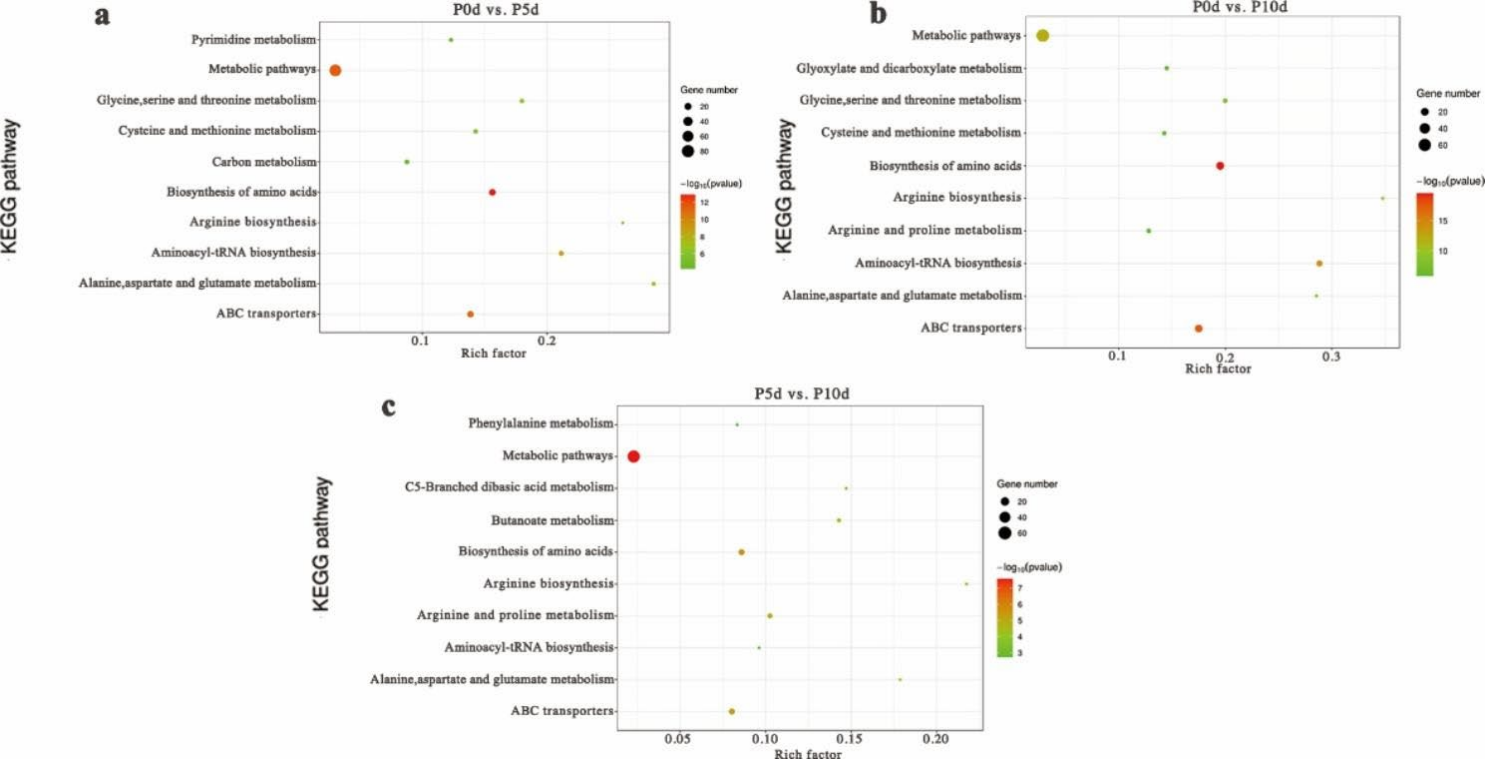
Top 10 KEGG pathway analysis of DAMs. (**a**) P0d vs. P5d; (**b**) P0d vs. P10d; (**c**) P5d vs. P10d

We performed KEGG enrichment analysis of the DAMs using several comparisons to identify the main metabolic pathways (Fig. 5 and Supplementary Figure S1-S5). The results showed that DAMs were significantly enriched in metabolic pathways in all groups. In addition to metabolic pathways, amino acid synthesis pathway and ABC transport factor were also one of the main pathways significantly enriched during imbibition, especially in the early stage of imbibition. In the different germination processes, we found that 2-oxocarboxylic metabolism tended to be significantly enriched in the comparison groups on 2days vs. 4days. Amino acid and ABC transporters were also significantly enriched in the comparison groups at the early and late germination stages. At different temperatures, most KEGG pathways were enriched in metabolic pathways, amino acid pathways and ABC transporters, and a small number of comparison groups were enriched in the phenylalanine metabolism pathway.

The DAMs in the initial stage of pecan seed germination under three temperature conditions were analyzed for metabolic pathways, and 21 significantly metabolic pathways were enriched. Among them, cysteine and methionine metabolism, β-alanine metabolism, C5-branched dibasic acid metabolism, arginine and proline metabolism, pyrimidine metabolism, glutathione metabolism, glycine, serine and threonine acid metabolism and alanine, aspartic acid and glutamic acid metabolism. These 8 metabolic pathways appeared in 3 temperatures; one specific metabolic pathway, the pentose phosphate pathway, germinated at 15°C; when the seeds germinated at 30°C, biosynthesis of isoquinoline alkaloids, pantothenic acid and coenzyme A biosynthesis, lysine biosynthesis, tyrosine metabolism, butyrate metabolism, arginine and proline metabolism, and the TCA Cycle were enriched. Amino acid metabolism (aspartic acid, glutamic acid, arginine, alanine, etc.) may play a crucial role in promoting high-temperature germination of pecan seeds.

### WGCNA analysis

**Fig. 6:**
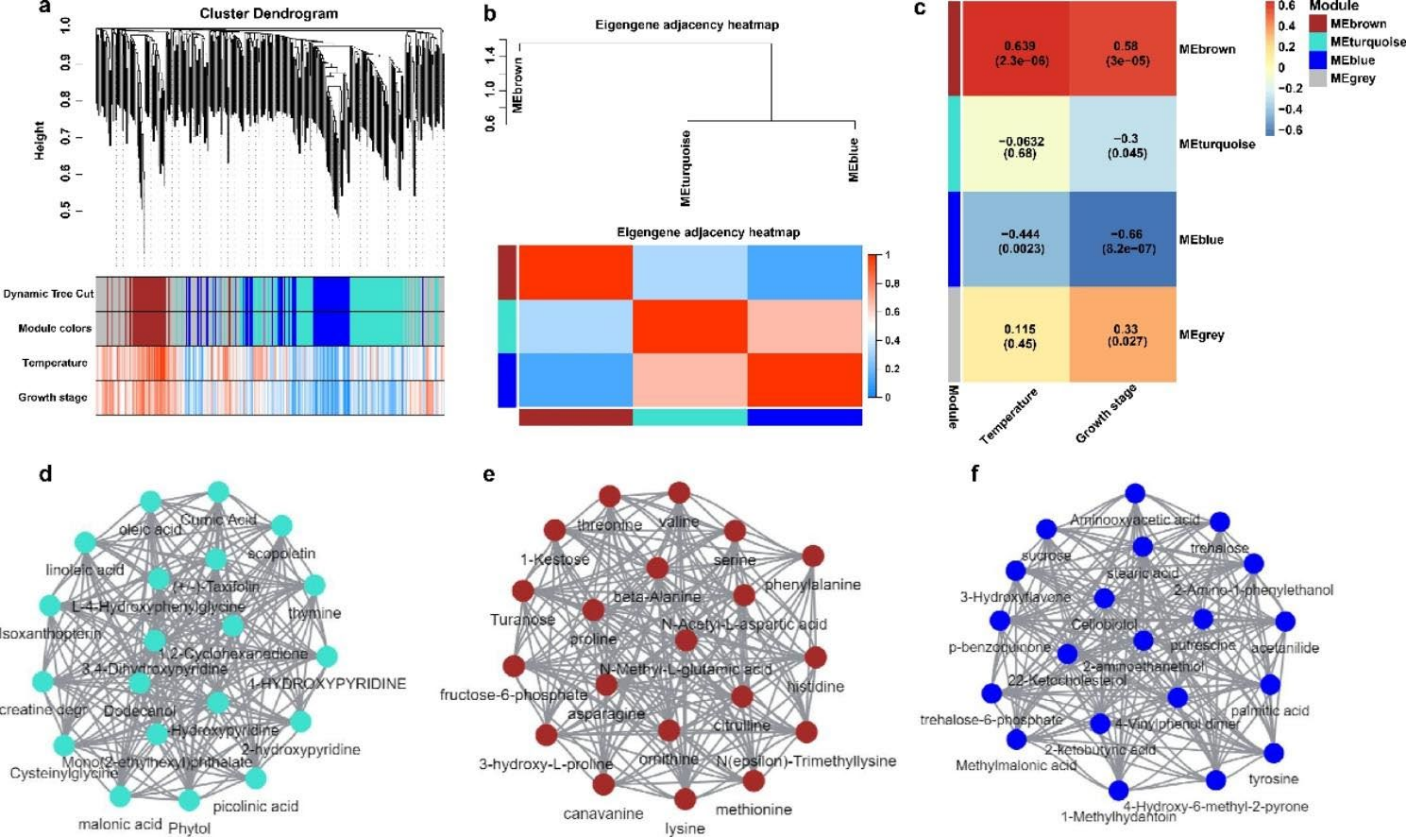
Hub metabolites related to germination temperature and stage of pecan seeds identified by WGCNA. (**a**) Clustering dendrogram of average network adjacency for the identification of metabolite co-expression modules. Clustering dendrogram of metabolites with dissimilarity based on topological overlap and assigned module colors; (**b**) Heatmap eigengenes connectivity. (**c**) Correlation between the WGCNA module, germination temperature, and growth stage of pecan seeds. The rows correspond to the modules, and the columns correspond to the features. The darker the red or blue color in the cell, the higher the positive or negative correlation, respectively. The number in each cell represents the corresponding correlation and *P*-value. (**d-f**) Network diagram of hub metabolites in three modules with high correlation: (**d**) turquoise module; (**e**) brown module; (**f**) blue module

We preformed WGCNA to calculate the relationship between metabolites and antioxidant activity. Metabolites were divided into three modules, and those that did not belong to these modules are shown in gray (Fig. 6a). The brown and blue modules were highly correlated with temperature and germination processes. Several identified metabolites may be responsible for the different temperatures of pecan seeds. Based on the WGCNA data, the hub metabolites of each module were further identified, and the correlation between each module and the germination temperature and stage of pecan was analyzed (Fig. 6d-f). Hub metabolites were found to be significantly related to temperature and temperature processes in these two modules. In the brown module, most hub metabolites were related to amino acids including, valine, threonine, serine, lysine, citrulline, 3-hydroxy-proline, phenylalanine, methionine and ornithine. Thus, it can be inferred that amino acids play an important role in pecan seed germination. This result has also been observed in other seed germination processes, such as in legume and *Arabidopsis* seeds [25, 26]. We found acids in both the turquoise and blue modules, including palmitic acid, oleic acid, linoleic acid, malonic acid, and palmitic acid. Pecan is an oil crop with fat and protein being the main storage substances. During seed germination, stored substances are transformed, transported, utilized, and synthesized into new tissues to provide energy and nutrients for seed germination. Fat mainly accumulates in the form of triacylglycerol and partly accumulates as glycerol and fatty acids [27]. From the initial stage of seed development to the transitional stage, the main form of fat is fatty acids; in the final stage of development, the main form of fat is triacylglycerol [27]. Triacylglycerols are stored in oleosomes and are broken down for energy during seed germination. During oilseed development, many storage proteins accumulate and are thought to provide nitrogen sources for seed germination [28].

Sugars are primary metabolites produced during seed germination. Starch (high molecular storage sugar) decomposes into small molecule sugar under the action of amylase, which is used for the cell synthesis and as a substrate for respiration to be decomposed and consumed [29]. During seed germination, soluble sugars are in a dynamic equilibrium [30]. Through WGCNA, we found that sugars such as sucrose, trehalose and cellobiotol were the main metabolites produced during pecan germination, indicating that sugars played an important role in seed germination. Raffinose is beneficial for the radicle to break through the seed coat [31] and early in wheat germination, and is transferred from the endosperm to the embryo, which is consistent with the results of this study [32].

During seed germination, the stored protein is continuously decomposed and the amino acids formed become raw materials for new protoplasts [33]. Studies have shown that soluble proteins originally used for storage are first degraded into amino acids during the germination preparation of pecan seeds. After the metabolism related to the germination of pecan seeds has begun, new proteins are synthesized in large quantities for radicle and embryonic growth and development. Although the content of free amino acids in the seeds showed certain differences among the three temperatures, the trend of change was basically the same. This indicates that the metabolism of proteins to free amino acids is vigorous under high-temperature conditions [34]. Simultaneously, new protein bodies, which are the main storage proteins, are synthesized and accumulated during seed germination. When seeds begin to germinate, the protein bodies begin to decompose and are consumed. When seed cotyledons emerges, almost all protein bodies are completely disintegrated [35].

Another way for seeds to store nutrients is to convert them into fats. With the participation of lipase, fat can be hydrolyzed to produce glycerol and fatty acids, which can be further converted into sugars. Generally, in the early stages of germination, the amount of decomposed fat is relatively low. The crude fat content in *Pinus* seeds during germination showed that the change in crude fat in the early stage was small, and the seeds began to use a large amount of fat after breaking through the seed coat, degrading and generating a large amount of sugar [36]. The germination of pistachio (*Pistacia vera*) [37], European hazelnut (*Corylus avellana*) [38] and other seeds also showed a similar results. When the radicle breaks through the seed coat, the fat can be hydrolyzed to glycerol and fatty acids by lipase, and the fatty acids are further converted into sugars [39].

## Conclusions

In this study, using non-targeted metabolomics, we identified 349 metabolites in pecan seeds at three different temperatures and found that amino acids are the most important metabolites in the germination process of pecan seeds. Using WGCNA, we identified characteristic metabolites of pecan seeds at different temperatures. Based on the metabolomics data, we found that the metabolism of amino acids, sugars, carbohydrates, and other substances and energy during the germination of pecan seeds was relatively active, and the germination effect was optimal at a temperature of 30 °C. Utilization of nutrients inside the pecan ensured that it maintained a more active metabolic process during germination to achieve the seedling establishment process.

## Electronic supplementary material

Below is the link to the electronic supplementary material.

**Supplementary files**: **Table S1** The seed moisture contents and germination rate of pecan seeds germinated in 15, 25 and 30°C. **Figure S1** Top 10 KEGG pathway analysis of DAMs in 2-day and 4-day germination process. (a, c and e) 2-day; (b, d and f) 4-day. **Figure S2** Top 10 KEGG pathway analysis of DAMs in 6-day and 12-day germination process. (a, c and e) 6-day; (b, d and f) 12-day. **Figure S3** Top 10 KEGG pathway analysis of DAMs in 15°C germination process. **Figure S4** Top 10 KEGG pathway analysis of DAMs in 25°C germination process. **Figure S5** Top 10 KEGG pathway analysis of DAMs in 30°C germination process.

**Table S2** Metabolites at 15°C

**Table S3** Metabolites at 25°C

**Table S4** Metabolites at 30°C

## Data Availability

The datasets generated and/or analyzed during the current study are not publicly available due the paper unpublished but are available from the corresponding author on reasonable request.
