# Supplementary material for: Metabolomics based on GC-MS revealed hub metabolites of pecan seeds germinating at different temperatures
Source: BMC Plant Biol. 2023 Apr 10;23:192. doi: 10.1186/s12870-023-04209-8 (PMC10084692; doi:10.1186/s12870-023-04209-8)

**Supplementary files**

**Table S1 The seed moisture contents and germination rate of pecan seeds germinated in 15, 25 and 30℃**

**Table S2 Metabolites at 15℃**

**Table S3 Metabolites at 25℃**

**Table S4 Metabolites at 30℃**

**Figure S1 Top 10 KEGG pathway analysis of DAMs in 2-day and 4-day germination process. (a, c and e) 2-day; (b, d and f) 4-day.**

**Figure S2 Top 10 KEGG pathway analysis of DAMs in 6-day and 12-day germination process. (a, c and e) 6-day; (b, d and f) 12-day.**

**Figure S3 Top 10 KEGG pathway analysis of DAMs in 15℃ germination process.**

**Figure S4 Top 10 KEGG pathway analysis of DAMs in 25℃ germination process.**

**Figure S5 Top 10 KEGG pathway analysis of DAMs in 30℃ germination process.**

**Table S1 The seed moisture contents and germination rate of pecan seeds germinated in 15, 25 and 30℃**

| Temperature/℃ | Time/d | Water content/% | Germination rate% |
| --- | --- | --- | --- |
| Room temperature（20） | 0 | 4 | 0 |
|  | 5 | 23 | 0 |
|  | 10 | 26 | 0 |
| 15 | 12 | 25 | 0 |
|  | 14 | 27 | 0 |
|  | 16 | 25 | 0 |
|  | 22 | 29 | 0 |
| 25 | 12 | 24 | 0 |
|  | 14 | 24 | 0 |
|  | 16 | 25 | 0 |
|  | 22 | 27 | 4 |
| 30 | 12 | 24 | 0 |
|  | 14 | 27 | 21 |
|  | 16 | 30 | 38 |
|  | 22 | 39 | 100 |

**Figure S1**


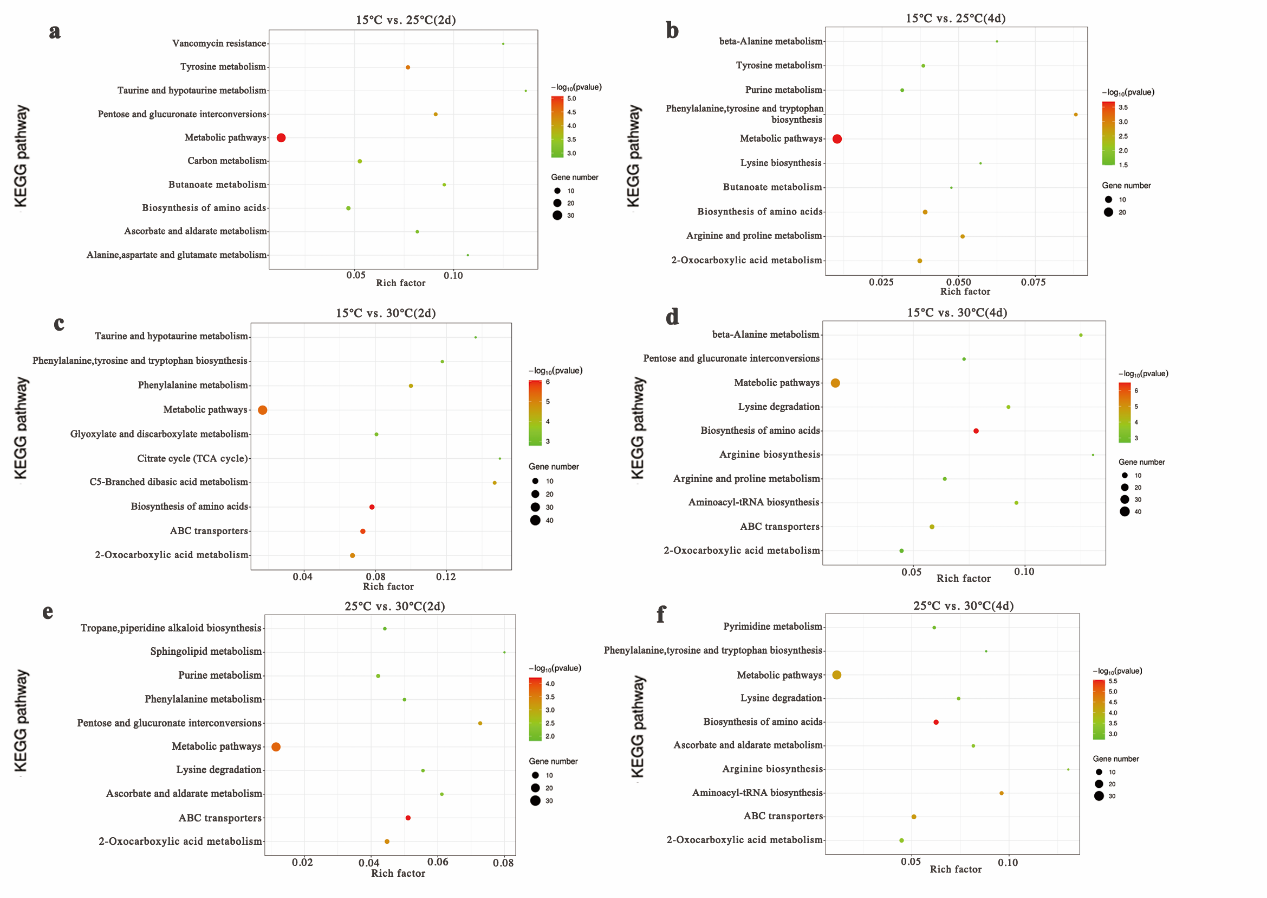


**Figure S2**


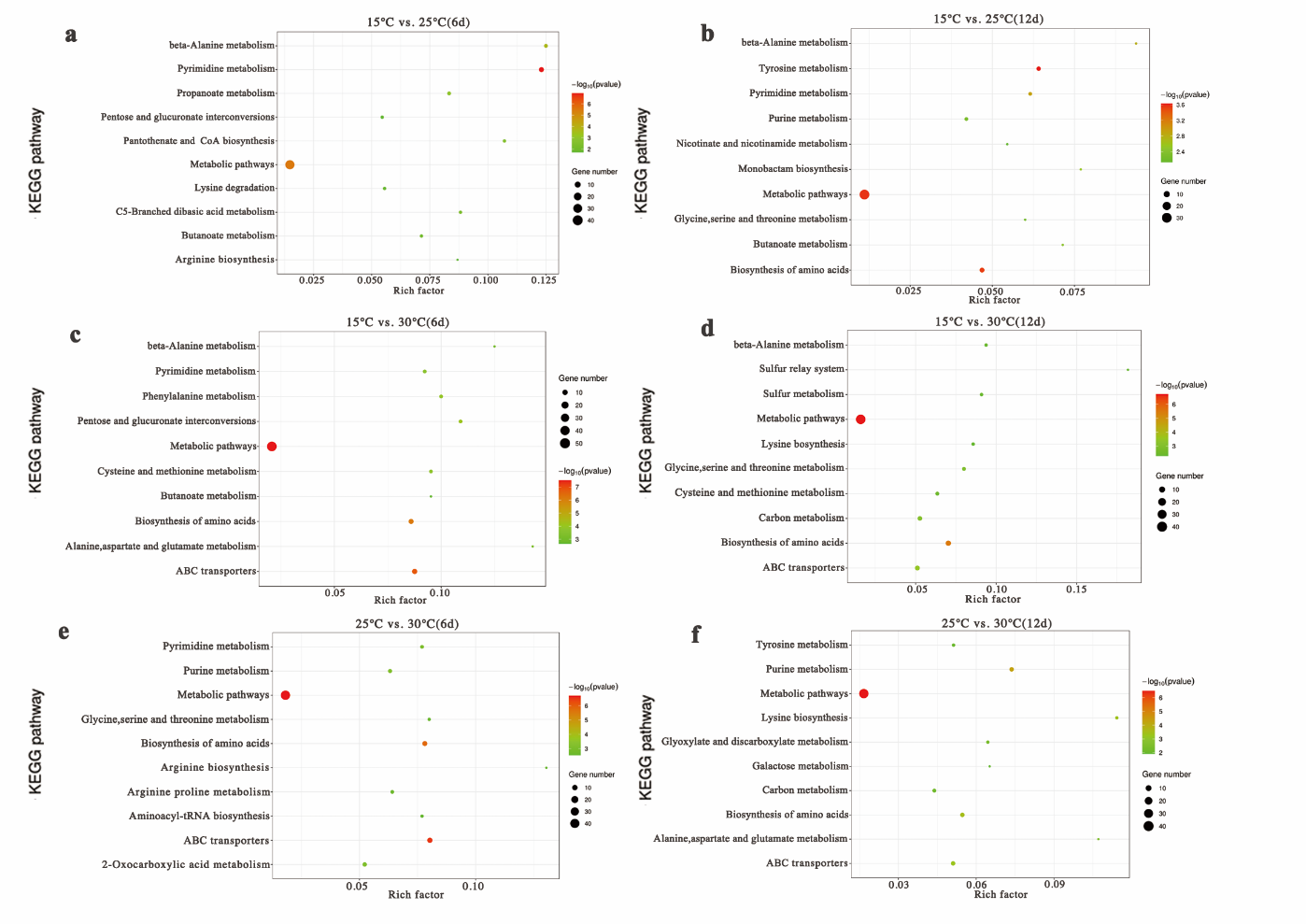


**Figure S3**


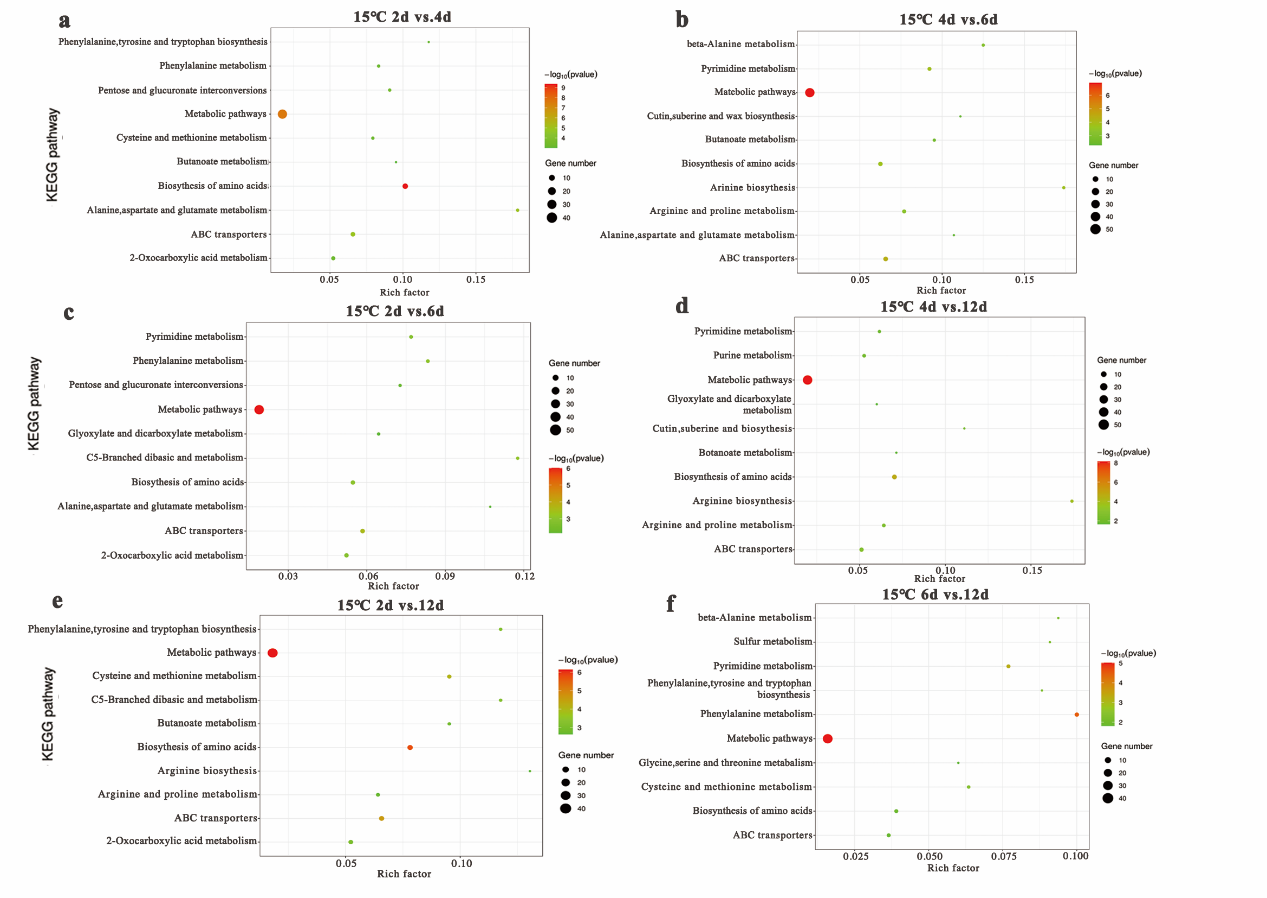


**Figure S4**


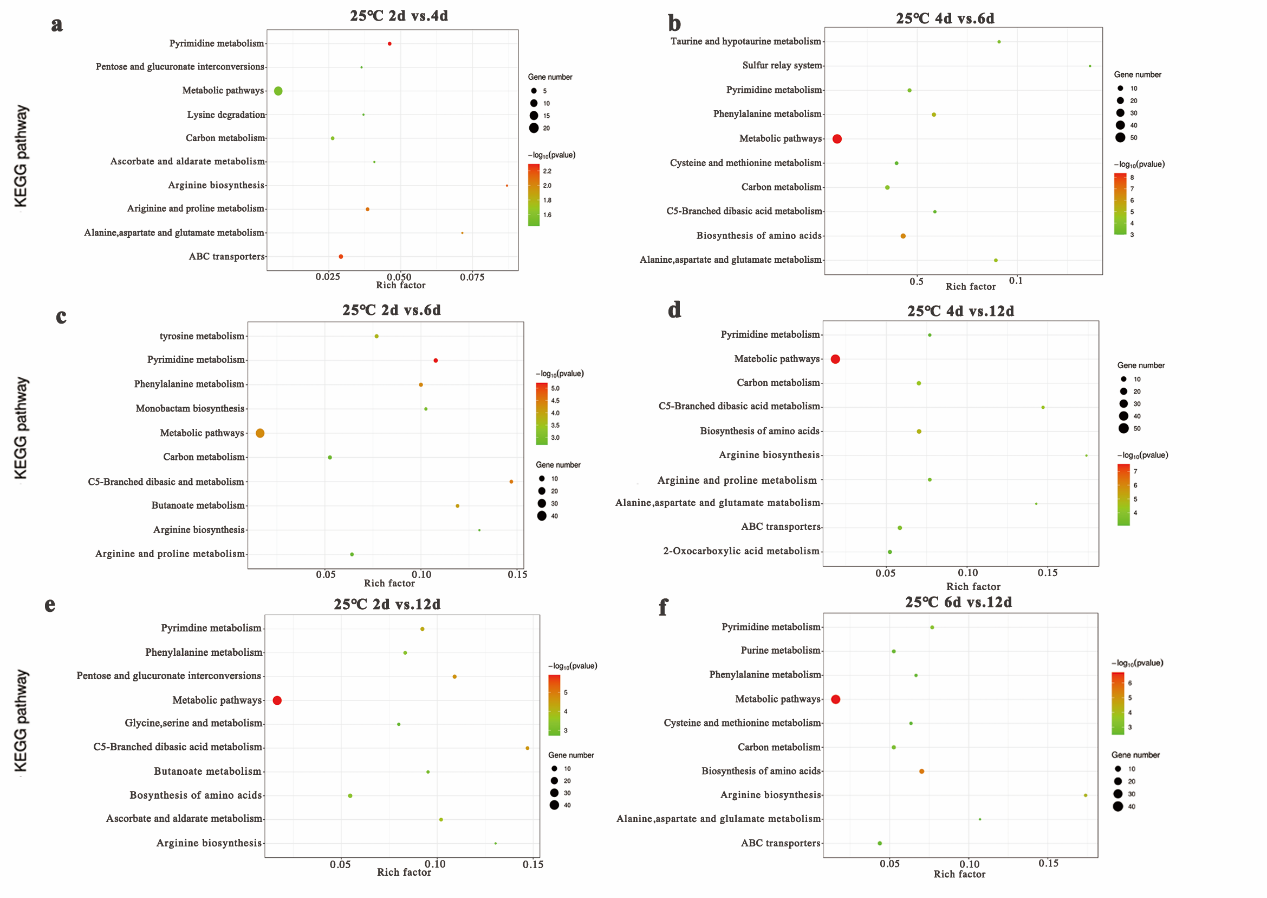


**Figure S5**


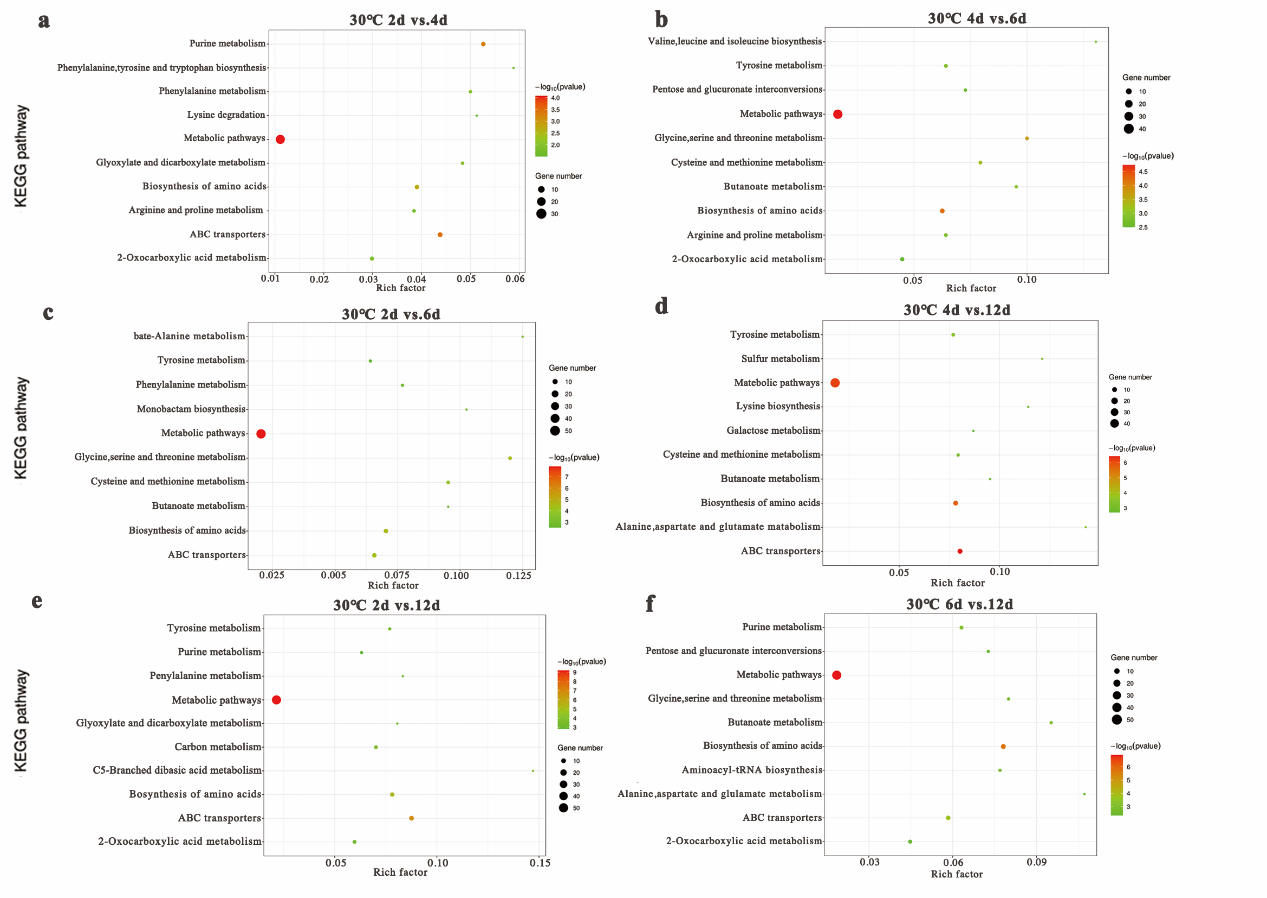

Supplement: Supplementary file 1 — Supplementary files: Table S1 The seed moisture contents and germination rate of pecan seeds germinated in 15, 25 and 30°C. Figure S1 Top 10 KEGG pathway analysis of DAMs in 2-day and 4-day germination process. (a, c and e) 2-day; (b, d and f) 4-day. Figure S2 Top 10 KEGG pathway analysis of DAMs in 6-day and 12-day germination process. (a, c and e) 6-day; (b, d and f) 12-day. Figure S3 Top 10 KEGG pathway analysis of DAMs in 15°C germination process. Figure S4 Top 10 KEGG pathway analysis of DAMs in 25°C germination process. Figure S5 Top 10 KEGG pathway analysis of DAMs in 30°C germination process. [file 12870_2023_4209_MOESM1_ESM.docx]
